# Metal Ion Release from Engineered Stone Dust in Artificial Lysosomal Fluid—Variation with Time and Stone Type

**DOI:** 10.3390/ijerph18126391

**Published:** 2021-06-12

**Authors:** Preeti Maharjan, Joseph Crea, Michael Tkaczuk, Sharyn Gaskin, Dino Pisaniello

**Affiliations:** Adelaide Exposure Science and Health, School of Public Health, University of Adelaide, Adelaide, SA 5005, Australia; maharjanpreety@gmail.com (P.M.); joseph.crea@adelaide.edu.au (J.C.); michael.tkaczuk@adelaide.edu.au (M.T.); sharyn.gaskin@adelaide.edu.au (S.G.)

**Keywords:** engineered stone, artificial stone, metal ion, biosolubility, silicosis, artificial lysosomal fluid

## Abstract

Inhalational exposure to dust from engineered stone (ES), also known as artificial or composite stone, is associated with a specific disease profile, namely accelerated silicosis, and scleroderma. The pathogenic mechanisms are poorly understood, particularly the role of resin and metal ions. Metal ions are present in pigments and constituent minerals and may be considered potential contributors to toxicity. The aim of this preliminary study was to understand the solubility of ES-containing metals in artificial lysosomal fluid (ALF) simulating the acidic intracellular environment of the lung macrophage lysosome. Differences with respect to ES types and temporal release were explored. Ten ES products of variable colour and company origin were comminuted and assessed for four different metals, solubilized into ALF solutions at 1,2,4 and 8 weeks at 37 °C. There was significant variability in metal release, particularly with regard to iron and manganese, which could be correlated with the reflected brightness of the stone. A majority of the available Mn, Fe, Al and Ti was solubilized. Time trends for metal release varied with ES type but also with metal ion. The data suggest a high metal ion bioavailability once engulfed by lung macrophages. There is a need to investigate a wider range of ES dust and relate metal content to markers of ES toxicity.

## 1. Introduction

Engineered stone (ES), also known as artificial stone, is a composite material primarily used in kitchens and bathrooms as an alternative to granite and other natural stone. The exposure of workers breathing ES dusts during cutting, grinding, and abrasive polishing using power tools has been associated with accelerated silicosis, scleroderma, and upper respiratory diseases [1,2,3]. A high prevalence of a new form of silicosis associated with extensive use of ES materials was first reported in Israel in 2006 [4], following which, Spain, Italy, Australia, and United States have also reported cases of accelerated silicosis related to ES [5,6,7]. In addition to the increased incidence, epidemiological studies also suggest that the accelerated silicosis is associated with a comparatively rapid development of fibrosis, less exposure duration, a shorter latency period, and less visibility on X-ray and CT scan [7,8]. Although there has been significant progress in reducing inhalation dust hazards in Australian workplaces, preventable occupational lung diseases such as silicosis have re-emerged [5,9]. There is uncertainty about the incidence and prevalence of accelerated silicosis in Australia, partly due to the absence of a national dust disease registry, and voluntary reporting by treating physicians. Voluntary notifications to the Thoracic Society of Australia and New Zealand’s Occupational Lung Diseases Special Interest Group identified seven ES-associated silicosis cases between 2011 and 2016 [5]. The median age of those reported cases was 43 years, and the median duration of exposure was only 7.3 years, representing an accelerated disease development among young workers. A formal screening program in Queensland identified silicosis in more than 12% of ES workers, a much higher prevalence than normally expected for silica-exposed workers. Many of these workers were deemed to require lung transplants [9]. The concerns about ES-associated silicosis and the comparatively young workers affected led to the development of the National Diseases Dust Taskforce in 2019 with the situation being viewed as a public health crisis [10].

In the case of ES, most benchtop fabricators are small businesses, and a lack of personal protective measures and engineering controls has been considered a plausible explanation for the occurrence of ES-related silicosis [3,5]. Engineered stones are significantly different from natural stones and differing compositions could be related to higher toxicity [3]. Apart from the high amount of crystalline silica, ES includes organic resin, pigments, and other minerals such as feldspar [11]. The evidence relating to the toxicological and chemical properties of ES dust appears sparse, particularly the potential role played by resin and metal ions in the development of lung disease and broader immunological effects [3,8]. Elemental components such as metal ions are considered important contributors to lung toxicity, primarily due to their ability to produce reactive oxygen species (ROS) [12,13]. Elements such as iron, chromium, and manganese may induce the overproduction of ROS causing oxidative stress leading to damage in cell components and ultimately cell malfunction and death. The few studies that have assessed the chemical composition of respirable ES dust suggest that hetero ions present in ES dust are potentially responsible for higher toxicity and reactivity [8,14,15,16]. Cohen [12] reviewed the pulmonary toxicology of a range of metals, including aluminium and transition metals such as manganese. Changes have been noted in alveolar macrophages for manganese as MnCl_2_ and nickel as NiCl_2._ More research is required, particularly for combinations of metals, for understanding intrapulmonary effects [12].

Element biosolubility studies are useful in assessing cell uptake evoking lung toxicity [17]. Simulated lung fluids (SLFs) simulating the interstitial and intracellular lung fluid environment have been used in several studies to assess the bioaccessibility of metallic constituents from vehicle dust, road exhaust, ultrafine airborne particulate matter, and high-temperature insulation wool [17,18,19]. To our knowledge, there is no reported study assessing the elemental bioaccessibility of ES dusts.

The aim of this preliminary study was to understand solubility of ES-containing metals in artificial lysosomal fluid (ALF) simulating the acidic intracellular environment of lung macrophage lysosomes. In particular, the biosolubility variability across ES types (manufacturer and colour), and the time trend of metal ion release are explored.

## 2. Materials and Methods

### 2.1. ES Dust Generation

Ten authentic ES samples were obtained from five different manufacturers, and were chosen on the basis of consumer popularity, colour and design. The organic content varied from 8.4–14.3% by weight. Each sample was initially cut using a wet diamond blade saw, and then crushed into small gravel-size pieces using a tungsten carbide jaw crusher in a specialized mineral processing facility in the University of Adelaide’s School of Physical Sciences. These were further comminuted using a tungsten carbide ring mill for 4 min, maintaining moderate temperatures, to generate fine dust containing respirable and inhalable size particles. The mid-point of the size distribution was 10–18 μm, as determined by wet sizing with a *Malvern Mastersizer* 2000. Cross-contamination between samples was avoided by thoroughly cleaning the jawcrusher and ring mill after and before each use. The handling of the comminuted ES was done in a fume cupboard as the dust was easily dispersed into the atmosphere. We used a real time aerosol photometer (TSI DustTrak, TSI Incorporated, Shoreview, MN, USA) to identify and monitor dusty aspects of the work.

### 2.2. Preparation of Artificial Lysosomal Fluids (ALF) and Its Extraction

Artificial lysosomal fluid (ALF) at pH 4.5, simulating the in vivo physiological condition of an acidic intracellular environment in lung cells, was used for bioaccessibility, as recommended by Pelfrene [20]. ALF was prepared following the composition used by Cannizzaro et al. [18]. Analytical grade chemicals and ultra-pure water were used throughout to avoid contamination.

Five grams of ES dust was mixed with 250 mL of ALF in a Schott bottle. ALF solution contains components such as pyruvate, citric acid, and glycine, which act as a growth media for many microorganisms; thus, 0.0002% formaldehyde was added to avoid microbial growth [18]. The sealed bottles containing a mixture of ES dust and ALF were placed in a shaker (*Ratek OM25* orbital/mixer incubator) at 37 °C and gently agitated at 80 rpm. Thirty-millilitre aliquots of ALF fluids were extracted periodically at one week, and two weeks, four weeks, and eight weeks of interaction with ES dust and was filled again with 30 mL of new ALF. The pH was noted at all time points for all ALF bottles with ES dust. A blank (250 mL ALF without any ES dust) was used for every batch as a quality control.

### 2.3. Determination of Metals and ES Reflected Brightness

Extracted 30 mL ALF aliquots from the 250 bottles were centrifuged in metal free centrifuge tubes at 4000 rpm for 15 min to settle any suspended particles if present. None of the aliquots had any visible residues at the bottom of the tube after centrifugation. The aliquots, including blanks, were forwarded to a commercial laboratory with a National Association of Testing Authorities accreditation to perform elemental analysis of metal ions such as iron (Fe), Manganese (Mn), Aluminum (Al) and Titanium (Ti). A standard method using inductively coupled plasma-mass spectrometry (ICP-MS) was used. Adjustments for dilution with fresh ALF were made in the reporting of metal ion concentrations. In initial experiments, a linear relationship was observed in metal ion concentrations for samples that had 1, 2, 4, and 10 g of solid. Thus, the values reported here are also normalized on a gram basis, i.e., concentrations are expressed as micrograms/litre per gram of solid.

Available metals (acid extractable) in the solid dust were determined by digestion with nitric and hydrochloric acid (1:1) for 1.5 h at 90–98 °C prior to analysis by ICP-AES (atomic emission spectroscopy) and ICP-MS.

The uncut ES samples were placed under normal overhead fluorescent lighting (4000 K) and reflected light was determined with a calibrated *Minolta NT-1* luminance meter. Values are expressed as candela per square metre.

### 2.4. Data Analysis

Descriptive statistics (with MS Excel) were used to summarise the concentration of metal ions for each stone type at each time point. The coefficient of variation was used to estimate the variability of the metal ion concentration results in replicate samples.

## 3. Results

Ten ES dusts (AES1, BES1, BES2, BES3, BES4, CES1, CES2, DES1, DES2 and EES1) from five different companies (prefixes A–E) were assessed.

Although 12 elements were determined, based on likely content in the ES, only four elements, aluminum, titanium, manganese, and iron are included here as the metal ion concentration of vanadium, arsenic, nickel, copper, chromium, and antimony were below the limit of detection. In addition, tungsten and cobalt were not included as these could be introduced as contaminants during stone dust generation using tungsten carbide [21].

All metal ion analyses conducted on blank ALF solutions at all time points were below the level of practical quantification. For Fe, Mn, Al and Ti, these were 10, 5, 10 and 1 μg/L for liquid samples. In the case of solid dust samples, these were 10, 1, 10 and 1 mg/kg.

In order to estimate the variability of quantified metal ion concentrations in ALF solutions, triplicate samples in separate bottles were run for two stone types (BES2 and BES4) across three time points (week 1, 2 and 4). The maximum coefficient of variation was 14.5% for Fe, 15.6% for Mn, 17% for Al and 22.8% for Ti.

### 3.1. Reflected Brightness of the Slab and Selected Chemical Characteristics in Unreacted ES Dust Samples

Table 1 shows the reflected brightness of the slab, as well as the presence and variabilities of selected metal elements, and main crystalline species before reaction with ALF. The reflected brightness correlated with the visual appearance of ES types, such that the dark-coloured stones had low values, while the light-coloured stones had the highest values. In addition, the ES stones with low brightness (AES1, BES2, and DES1) had comparatively higher amounts of iron and manganese. Manganese values tended to follow iron values.

### 3.2. Release of Metal Ions in Artificial Lysosomal Fluid

**Figure 1:**
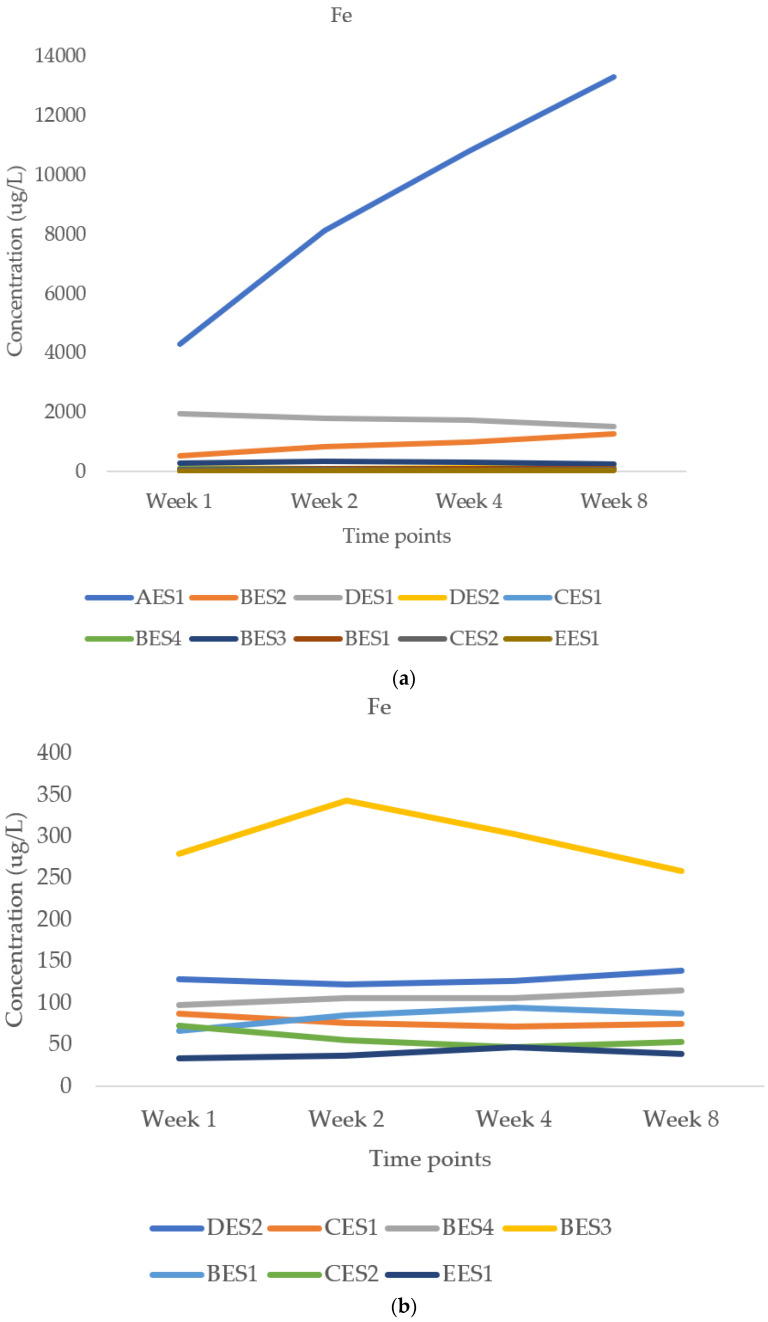

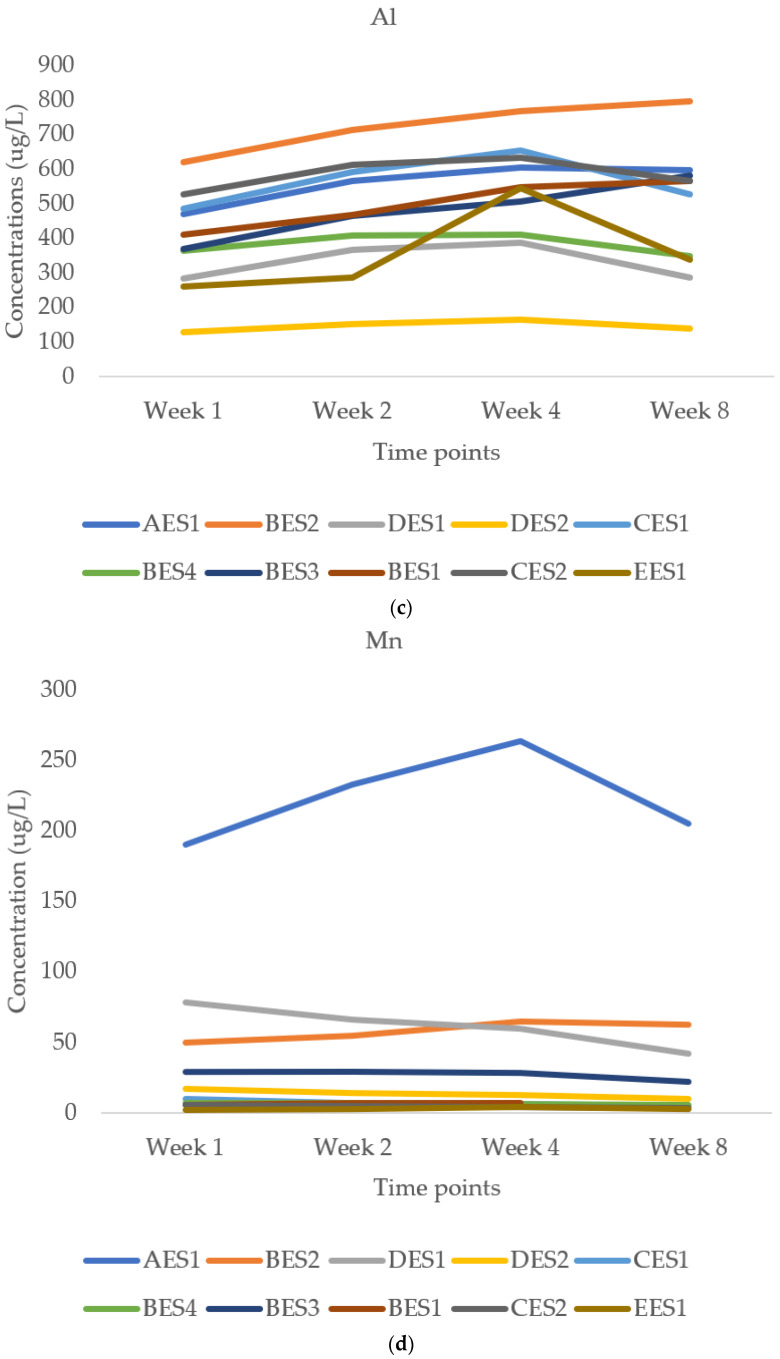

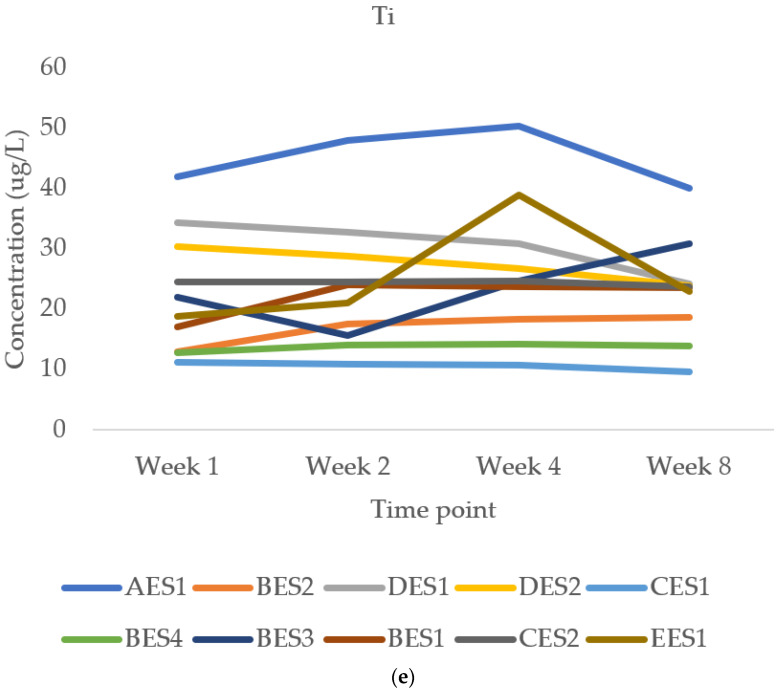
(**a**) Iron release (μg/L per gram of solid) from various ES at different timepoints; (**b**) Iron release (μg/L per gram of solid) at different time points, excluding AES1, BES2 and DES1; (**c**) Aluminium release (μg/L per gram of solid) from various ES at different timepoints; (**d**) Manganese release (μg/L per gram of solid) for various ES at different time points; (**e**) Titanium release (μg/L per gram of solid) for various ES at different time points.

There were significant differences in the release of Fe, Al, Mn and Ti, depending on the stone type. These tend to reflect the metal amounts inherent in the dust (Table 1). Figure 1a,b relate to iron. Figure 1b excludes AES1, BES2 and DES1, which had high levels of iron. Figure 1c–e relate to Al, Mn and Ti respectively.

#### 3.2.1. Iron

There is significant variability in absolute terms. As can be seen from Figure 1a, AES1 had the highest value of Fe release and the values increased steadily from week 1 to week 8. It is interesting that this sample had 0.8% crystalline magnetite as determined by quantitative X-ray diffraction, and that the dust can be attracted to a strong magnet. However, Fe release remained similar from week 1 and week 8 for other ES samples. These data suggest that iron is solubilised relatively rapidly, which was confirmed by several 3-day reaction experiments (not reported here).

#### 3.2.2. Aluminium

Aluminium was found to be a relatively abundant element released in ALF, which is to be expected as ES typically contains aluminosilicate minerals, such as feldspars. Figure 1c shows moderate variability in absolute terms and that aluminium release increased slightly from week 1 to week 8 for most of the ES samples.

#### 3.2.3. Manganese and Titanium

Figure 1d shows that there is significant variability in the manganese release in absolute terms, but the time trend is relatively flat. Figure 1e shows less variability for titanium in absolute terms and similar flatness in terms of time trend. Titanium can be present as titanium dioxide, used as a white pigment.

#### 3.2.4. Correlation of Reflected Brightness with Metal Ion Release

**Figure 2:**
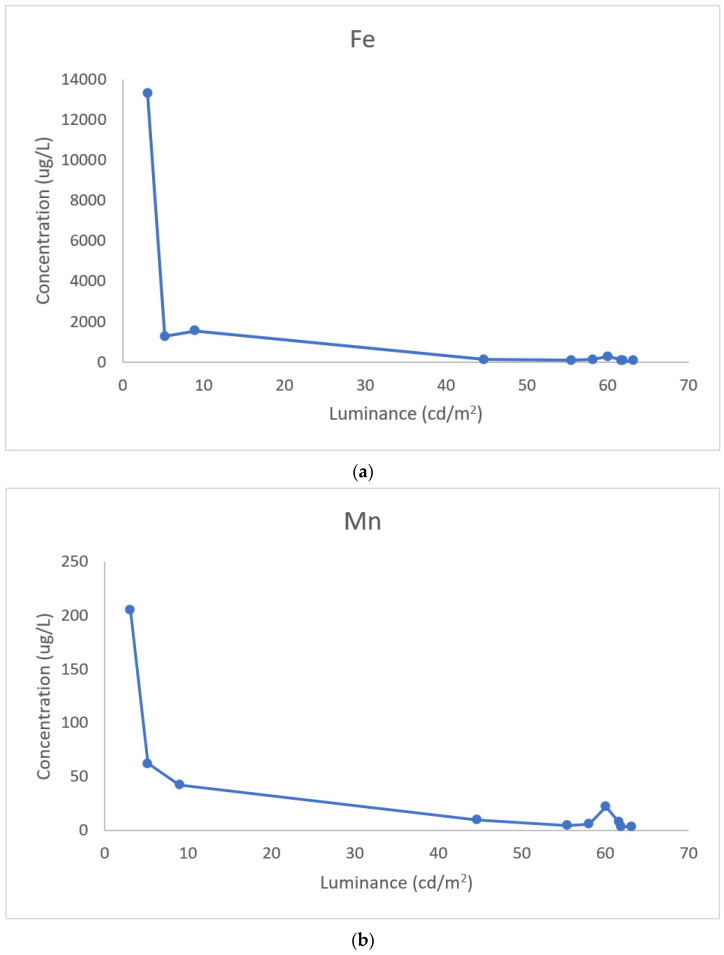
(**a**) Iron release (μg/L per gram of solid) versus reflected brightness. (**b**) Manganese release (μg/L per gram of solid) versus reflected brightness.

Figure 2a,b show an inverse relationship between reflected brightness and the maximum levels of iron and manganese release. This is consistent with the data in Table 1 pertaining to acid-extractable metal content of the solid dust.

### 3.3. Degree of Solubilisation of Metals in Artificial Lysosomal Fluid

The degree of metal solubilisation in ALF varied by sample type and metal. The proportion of metal released, relative to the amount available, was calculated as (maximum metal ion concentration (μg/L) × 0.25 L)/available metal in the dust (μg/g).

Table 2 shows that the proportion was generally more than 50%. For example, iron solubilisation ranged from 39% in EES1 to 96% in BES3. In the case of aluminium, the aluminosilicate content appears to have decomposed.

## 4. Discussion

This is the first study reporting ES metal ion release in ALF. The data indicate extensive, and sometimes progressive, metal solubilization in acid environments (pH 4.5) corresponding to the intracellular environment of lung macrophage lysosomes. This is possibly due to chelation of the metals, driving the equilibrium towards soluble metal-organic moieties.

The presence of the metals is generally consistent with information in the safety data sheet (SDS) for the ES, including minerals such as rutile, magnetite, and hematite [11]. However, the information in SDSs is limited and does not provide proportionate information on iron or any other elemental constituents. In addition, the SDS are usually not specific for a stone type [11]. Thus, the users of the products are unaware of the amounts of metals in the base minerals or pigments. However, our data suggest that the reflected brightness may be an indication of iron and manganese content, and release (Table 1, Figure 2a,b).

Metals assessed in this study may enhance lung toxicity from crystalline silica [14,22,23]. Manganese and iron are essential cations for cell function in trace amounts. However, where there is imbalance, these are toxicogenic and damage lung cells and tissues [24]. The presence of excessive Fe and Mn may enhance production of reactive oxygen species (ROS), converting hydrogen peroxide into very reactive hydroxyl species, resulting in cell damage [25,26].

Unfortunately, there seem to be very limited data on normal intra-macrophage metal ion concentrations in the scientific literature. It is estimated that Fe concentrations in lysosomes are 1–5 μM (56–279 μg/L) compared with extracellular fluid of 15–20 μM [27].

The source of various metals could be the mineral constituents, pigments, organic resins, and stone processing activities [14]. Precise information on minerals, pigments and resins used for ES manufacture is commercially sensitive and the elements could vary depending on the source of raw materials used. A source of Fe and Mn could possibly be the inorganic pigments. Titanium could be due to rutile, giving a white appearance. The stone processing activities in industrial stone workshops may introduce different elements such as Co, W and Fe [16,21]. For example, Co and W could be introduced through cutting and grinding of ES slabs using tungsten carbide. A study comparing parent and processed ES dust found that there can be significant chemical variability between parent and processed dust. The variation was found to relate to the type of processing such as dry cutting or wet cutting [16]. Another source of element contamination in ES is the polyester resin binder, although the amount of metal catalyst is very small [28].

This study has a number of strengths. It examined products from multiple suppliers, and examined metal ion release into ALF over an extended time period. The dust was generated by moderate temperature comminution of authentic slab samples, avoiding any decomposition.

There are some limitations. This preliminary study mainly focused on elemental analysis and there was no speciation of the metals. Comprehensive physicochemical characterisation of the ES dust, including electron microscopy was not done. Further information on physicochemical characteristics would be helpful in further understanding pathogenicity. Toxicology experiments can be coupled with the information of metal ion release. These include cellular studies and hemolysis assays of ES that have significantly different metal ion content. In this case, the role of metals in hemolysis and subsequent fibrosis can be explored [29,30].

These findings may be useful in modifying the ES composition of these ES to make safer products.

## 5. Conclusions

This study demonstrated the substantial variability in potential metal ion release from ES dust in a simulated lung cell environment. There were significant differences in metal ion release, depending on the ES type, particularly iron release. Time trends for metal release also varied with ES type but also metal ion. With different varieties of ES available in market and data suggesting variability of metal release with ES, there is a need for further investigation of the toxicological properties of ES dust.

## Figures and Tables

**Table 1:** Physico-chemical characteristics of stone types: Brightness of original ES samples, metal concentrations in dust and main crystalline species.

Stone Type	Brightness (cd/m^2^)	Fe (mg/kg)	Mn (mg/kg)	Al (mg/kg)	Ti (mg/kg)	Main Crystalline Mineral Species *
AES1	3.1	3800	63	150	9	89% quartz; 3% albite; 0.8% magnetite
BES1	62	40	1	230	7	56% quartz, 24% cristobalite, 2% albite
BES2	5.2	710	23	250	4	92% quartz
BES3	60	90	7	160	7	19% quartz, 47% cristobalite, 6% albite
BES4	58	50	3	190	6	23% quartz, 43% cristobalite, 7% albite
CES1	56	50	3	330	6	98% quartz, 2% rutile #
CES2	39	40	2	400	10	96% quartz, 4% rutile #
DES1	9.0	565	21	140	8	90% quartz
DES2	45	80	3	60	7	76% quartz, 23% cristobalite #
EES1	63.2	30	1	240	9	99% quartz #

* determined by X-ray Diffraction; # semi-quantitative.

**Table 2:** Available metal in dust and percentage solubilisation of metals in ALF.

Metal		AES1	BES1	BES2	BES3	BES4	CES1	CES2	DES1	DES2	EES1
Fe (mg/kg or μg/g))	Available metal in solid dust	3800	40	710	90	50	50	40	565	80	30
Fe (μg/L)	Maximum metal release	13,300	95	1257	344	116	87	73	1940	139	47
	% of metal solubilised in ALF	87	59	44	96	58	44	46	86	43	39
Mn (mg/kg)	Available metal in solid dust	63	1	23	7	3	3	2	21	3	1
Mn (μg/L)	Maximum metal release	264	7	65	29	7	10	5	79	17	4
	% of metal solubilised in ALF	- *	- *	71	- *	58	83	63	94	- *	- *
Al (mg/kg)	Available metal in solid dust	150	230	250	160	190	330	400	140	60	240
Al (μg/L)	Maximum metal release	604	568	796	583	410	654	635	389	164	545
	% of metal solubilised in ALF	100	62	80	91	54	50	40	69	68	57
Ti (mg/kg)	Available metal in solid dust	9	7	4	7	6	6	10	8	7	9
Ti (μg/L)	Maximum metal release	50	24	19	31	14	11	25	35	30	39
	% of metal solubilised in ALF	- *	86	- *	- *	58	46	63	- *	- *	- *

* could not be reliably determined due to low initial concentration.

## Data Availability

The data presented in this study are available on request from the corresponding author.
